# Dynamic Imprinting of the Treg Cell-Specific Epigenetic Signature in Developing Thymic Regulatory T Cells

**DOI:** 10.3389/fimmu.2019.02382

**Published:** 2019-10-11

**Authors:** Susanne Herppich, Aras Toker, Beate Pietzsch, Yohko Kitagawa, Naganari Ohkura, Takahisa Miyao, Stefan Floess, Shohei Hori, Shimon Sakaguchi, Jochen Huehn

**Affiliations:** ^1^Department Experimental Immunology, Helmholtz Centre for Infection Research, Braunschweig, Germany; ^2^Laboratory of Experimental Immunology, World Premier International Research Center Immunology Frontier Research Center, Osaka University, Osaka, Japan; ^3^Laboratory for Immune Homeostasis, RIKEN Center for Integrative Medical Sciences, Yokohama, Japan; ^4^RIKEN Center for Integrative Medical Sciences, Yokohama, Japan; ^5^Laboratory of Immunology and Microbiology, Graduate School of Pharmaceutical Sciences, The University of Tokyo, Tokyo, Japan

**Keywords:** Treg cell, Treg cell precursors, demethylation, epigenetic signature, IL-2, thymus, TSDR, Foxp3

## Abstract

Regulatory T (Treg) cells mainly develop within the thymus and arise from CD25^+^Foxp3^−^ (CD25^+^ TregP) or CD25^−^Foxp3^+^ (Foxp3^+^ TregP) Treg cell precursors resulting in Treg cells harboring distinct transcriptomic profiles and complementary T cell receptor repertoires. The stable and long-term expression of Foxp3 in Treg cells and their stable suppressive phenotype are controlled by the demethylation of Treg cell-specific epigenetic signature genes including an evolutionarily conserved CpG-rich element within the *Foxp3* locus, the Treg-specific demethylated region (TSDR). Here we analyzed the dynamics of the imprinting of the Treg cell-specific epigenetic signature genes in thymic Treg cells. We could demonstrate that CD25^+^Foxp3^+^ Treg cells show a progressive demethylation of most signature genes during maturation within the thymus. Interestingly, a partial demethylation of several Treg cell-specific epigenetic signature genes was already observed in Foxp3^+^ TregP but not in CD25^+^ TregP. Furthermore, Foxp3^+^ TregP were very transient in nature and arose at a more mature developmental stage when compared to CD25^+^ TregP. When the two Treg cell precursors were cultured in presence of IL-2, a factor known to be critical for thymic Treg cell development, we observed a major impact of IL-2 on the demethylation of the TSDR with a more pronounced effect on Foxp3^+^ TregP. Together, these results suggest that the establishment of the Treg cell-specific hypomethylation pattern is a continuous process throughout thymic Treg cell development and that the two known Treg cell precursors display distinct dynamics for the imprinting of the Treg cell-specific epigenetic signature genes.

## Introduction

CD4^+^ regulatory T (Treg) cells are crucial for the maintenance of self-tolerance. The continuous expression of the lineage-specification factor, Foxp3, endows these immunoregulatory cells with long-term stability and suppressive activity (1, 2). Accordingly, mutations in the *Foxp3* locus can result in an autoimmune and inflammatory syndrome in mice and humans [Scurfy and IPEX (immune dysregulation, polyendocrinopathy, enteropathy, X-linked) syndrome, respectively] (3–5). Although the induction and maintenance of Foxp3 expression are crucial for the lineage identity and functionality of Treg cells, Foxp3 expression as such is not sufficient to ensure complete Treg cell phenotypic and functional properties. For instance, retrovirally induced ectopic expression of Foxp3 in CD4^+^CD25^−^ conventional T cells could not induce the complete set of Treg cell-specific signature genes (6, 7). In line with this, *in vivo* disruption of the *Foxp3* gene by green fluorescent protein (GFP; *Foxp3*^*gfpko*^ mice) resulted in Foxp3^−^GFP^+^ cells still expressing several Treg cell-specific signature genes (8). To this end, it was shown that the CpG DNA demethylation at a set of Treg cell-specific epigenetic signature genes essentially but independently complements Foxp3 expression for entire Treg cell functionality and long-term lineage stability (9–12). Although significant progress has been made in understanding the importance of epigenetic imprinting on generating stable Treg cells, factors that initiate and drive this imprinting process are still incompletely understood.

Induction of Foxp3 expression and acquisition of the Treg cell-specific CpG hypomethylation pattern take place during thymic Treg cell development. It is assumed that the majority (~80%) of the Treg cell population originates from the thymus, termed thymus-derived Treg (tTreg) cells (13). The concurrent stimulation of the T cell receptor (TCR) and CD28 is viewed as the first step in a two-step model of thymic Treg cell development (14, 15). This model proposes that the first step is instructive for the up-regulation of the IL-2Rα subunit (CD25), resulting in the development of CD25^+^Foxp3^−^ Treg cell precursors (CD25^+^ TregP). Due to the expression of the high affinity IL-2 receptor, these cells are supremely sensitive to IL-2 and, at least a part of this precursor population can differentiate into CD25^+^Foxp3^+^ Treg cells in a second step upon stimulation with IL-2 without further need for TCR-derived signals (15). Accordingly, IL-2- or CD25-deficient mice display impaired tTreg cell development, exhibiting ~50% of normal Treg cell numbers among CD4 single-positive (SP) thymocytes (16, 17), and develop lymphoproliferative disease. Whether IL-2 signaling in CD25^+^ TregP is sufficient to drive epigenetic imprinting characteristic of mature tTreg cells is, however, not known. In addition to this model of Treg cell development, other studies indicate that Treg cells can also arise from CD25^−^Foxp3^+^ Treg cell precursors (Foxp3^+^ TregP) (18). Thus, it was proposed that TCR-CD28 co-stimulation and/or IL-15 might lead to the up-regulation of Foxp3 expression in CD4SP thymocytes (18, 19). Interestingly, Foxp3 was reported to be proapoptotic, and unless it is counterbalanced by IL-2 signals, Foxp3^+^ TregP undergo apoptosis (18). NF-κB is essential for the generation of both precursor populations. While IkB_NS_ and c-Rel together control the induction of Foxp3 expression in CD25^+^ TregP and Foxp3^+^ TregP, it was shown that c-Rel supports the induction of CD25 in both precursors (20–22). Recently, Owen et al. reported that CD25^+^ TregP and Foxp3^+^ TregP contribute almost equally to the generation of mature tTreg cells, despite showing distinct maturation kinetics and cytokine responsiveness. Additionally, the mature tTreg cells derived from the two precursors differed in their transcriptomes, their interactions with self-antigens and their TCR repertoire (23). In line with this, Foxp3^+^ TregP were shown to already possess a partially demethylated Treg-specific demethylated region (TSDR), while CD25^+^ TregP exhibited a completely methylated TSDR comparable to Foxp3^−^ CD4SP thymocytes (24). However, the dynamics of the imprinting and the establishment of the Treg cell-specific hypomethylation pattern as well as the involvement of IL-2 in the demethylation of the TSDR and other Treg cell-specific epigenetic signature genes in both precursor populations have not been investigated yet.

In this study, we assessed the dynamics of the imprinting of the Treg cell-specific epigenetic signature genes in tTreg cells and could demonstrate that CD25^+^Foxp3^+^ Treg cells show a progressive demethylation of most signature genes while maturing within the thymus. The two Treg cell precursors displayed distinct dynamics for the imprinting of the Treg cell-specific epigenetic signature genes, with Foxp3^+^ TregP already showing a partially established Treg cell-specific hypomethylation pattern. Intriguingly, we found that IL-2 mainly impacts the establishment and progression of TSDR demethylation with a more pronounced effect on Foxp3^+^ TregP when compared to CD25^+^ TregP. Thus, the two thymic Treg cell precursors differ substantially in the establishment of the Treg cell-specific hypomethylation pattern.

## Materials and Methods

### Mice

B6.*Foxp3*^tm1(CD2/CD52)Shori^ (Foxp3^hCD2^ reporter mice on C57BL/6 background), B6.SJL-*Ptprc*^*a*^*Pepc*^*b*^/BoyJ.*Foxp3*^tm1(CD2/CD52)Shori^ (CD45.1 congenic Foxp3^hCD2^ reporter mice on C57BL/6 background) and B6.SJL-*Ptprc*^*a*^*Pepc*^*b*^/BoyJ.*Foxp3*^tm1(CD2/CD52)Shori^-*Rag1*^tm1(GFP)Imku^ mice (CD45.1 congenic Foxp3^hCD2^xRag1^GFP^ reporter mice on C57BL/6 background) (25, 26) were bred and maintained at the central animal facility of the Helmholtz Center for Infection Research (HZI, Braunschweig, Germany), which provides state-of-the-art laboratory animal care and service. B6.*Foxp3*^tm2.1(EGFP/cre)Shori^.*Gt(ROSA)26Sor*^tm1Hjf^ mice (Foxp3^eGFPCre^xROSA26^RFP^ fate-mapping mice on C57BL/6 background) (26) were bread and maintained at the animal facility of the RIKEN Center for Integrative Medical Sciences (Yokohama, Japan). All mice were housed in barriers under specific pathogen-free (SPF) conditions in isolated, ventilated cages, and handled by personnel appropriately trained as well as dedicated animal care staff to assure the highest possible hygienic standards and animal welfare in compliance with German, European and Japanese animal welfare guidelines. According to the German Animal Welfare Act (§4, section Discussion) sacrifizing animals solely to remove organs for scientific purposes is notified to the competent authority. This study was carried out in accordance with the principles of the Basel Declaration as well as recommendations as defined by FELASA (Federation of European Laboratory Animal Science Associations), the German animal welfare body GV-SOLAS (Society for Laboratory Animal Science), and the Institutional Animal Care at RIKEN using approved protocols. All mice were used at the age of 4–9 weeks.

### Antibodies and Flow Cytometry

Cell suspensions were labeled directly with the following fluorochrome-conjugated anti-mouse antibodies purchased from either BioLegend or eBioscience: CD4 (RM4-5), CD8α (53–6.7), CD24 (M1/69), CD25 (PC61.5), CD73 (TY/11.8), and anti-human CD2 (RPA-2.10). To block the Fc-receptors, the staining mix always contained unconjugated anti-FcRγIII/II antibody (BioXcell; final concentration 10 μg ml^−1^). For exclusion of dead cells, 4′,6-Diamidine-2′-phenylindole dihydrochloride (Merck) was used. Stained cells were assessed by LSRFortessa™ or FACSCanto™ II flow cytometer (BD Biosciences) and data were analyzed with FlowJo^®^ software (TreeStar).

### Cell Sorting

Single-cell suspensions from thymi were depleted of APC-labeled CD8α^+^ cells using anti-APC microbeads (Miltenyi Biotec) and the autoMACS^®^ Pro Separator (Miltenyi Biotec). The negative fraction was stained with fluorochrome-conjugated anti-mouse antibodies and subsets of CD4SP thymocytes were sorted using a FACSAria™ or a FACSAria™ Fusion (BD Biosciences).

### Cell Culture

Sorted CD4SP thymocyte subsets were cultured at 37°C and 5% CO_2_ for 3 or 5 days. For this purpose, 5 × 10^4^ cells/well were placed within 100 μl of Roswell Park Memorial Institute medium (RPMI, Gibco) completed with 10% FCS, 50 U ml^−1^ penicillin, 50 U ml^−1^ streptomycin, 25 mM HEPES, 1 mM sodium pyruvate (all Biochrom AG), 50 μM β-mercaptoethanol (Gibco) containing 50 ng ml^−1^ recombinant mouse IL-2 (R&D Systems) into a round-bottom 96-well plate (Sarstedt).

### DNA Methylation Analysis

Genomic DNA was isolated from sorted CD4SP thymocyte subsets using the DNeasy^®^ Blood & Tissue Kit (Qiagen) and concentrated using the DNA Clean & Concentrator Kit (Zymo Research), both following the manufacturers' protocols. The DNA concentration was quantified with a Nanodrop 1000 spectrophotometer (Peqlab). Methylation analysis of the TSDR and other Treg cell-specific epigenetic signature genes was performed using bisulfite sequencing as described before (27). Exclusively, cells from male mice were used for the methylation analysis. For each CD4SP thymocyte subset, cells were pooled from 4 to 6 independent experiments to reach sufficient cell numbers for the methylation analyses.

### Statistical Analysis

The GraphPad Prism software v7.0 (GraphPad) was used to perform all statistical analyses. Data are presented as mean ± standard deviation (SD). For comparison of unmatched groups, two-tailed Mann-Whitney test was applied and the *p*-values were calculated with long-rank test (Mantel-Cox). If comparing more than two groups Kruskal-Wallis-Test with Dunn's test was used. A *p*-value below 0.05 was considered as significant (^*^p < 0.05; ^**^p < 0.01; ^***^p < 0.001; ^****^p < 0.0001; ns, not significant).

## Results

### Newly Generated Treg Cells Progressively Mature Within the Thymus

**Figure 1:**
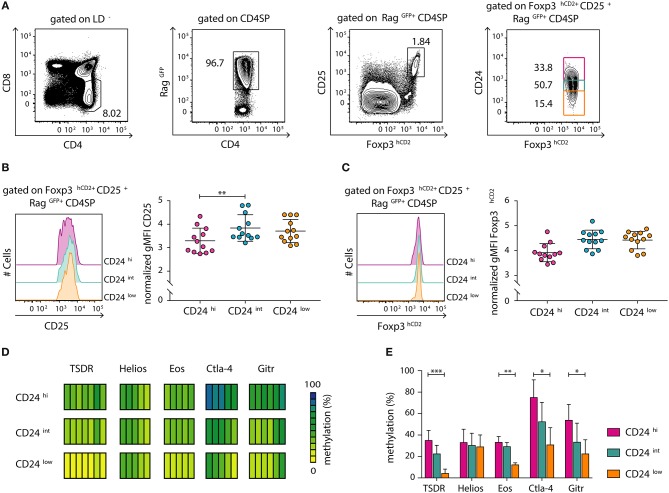
Newly generated CD25^+^Foxp3^hCD2+^ Treg cells progressively mature within the thymus. Thymocytes were isolated from Foxp3^hCD2^xRag1^GFP^ reporter mice and analyzed by flow cytometry or prepared for subsequent methylation analysis. **(A)** Representative dot plots show the gating of CD4SP thymocytes among living cells (LD^−^, LIVE/DEAD^−^), newly generated Rag^GFP+^ cells among CD4SP thymocytes, CD25^+^Foxp3^hCD2+^ Treg cells among Rag^GFP+^CD4SP thymocytes, and CD24^hi/int/low^ cells among CD25^+^Foxp3^hCD2+^Rag^GFP+^CD4SP thymocytes. Numbers specify frequencies of cells in indicated gates. **(B,C)** (Left) Representative histograms depict the expression of CD25 **(B)** and Foxp3^hCD2^ **(C)** among CD24^hi/int/low^ subsets of CD25^+^Foxp3^hCD2+^Rag^GFP+^CD4SP thymocytes. (Right) Scatter plots summarize the data from three independent experiments and bars indicate mean ± SD. Each symbol represents an individual mouse. The significance was calculated using Kruskal-Wallis with Dunn's test (^**^p < 0.01). Data are given relative to the maximal span (x_max_-x_min_) per experiment. **(D,E)** Genomic DNA was isolated from sorted CD24^hi/int/low^ subsets of CD25^+^Foxp3^hCD2+^Rag^GFP+^CD4SP thymocytes for analysis of the methylation status of TSDR, *Helios, Eos, Ctla-4*, and *Gitr*. Cells were pooled from four independent sorts. **(D)** Each bar represents an individual CpG motif. Percentage of methylation is color-coded according to the scale. **(E)** Bar graph summarizes changes in the CpG methylation ratio of the Treg cell-specific epigenetic signature genes in CD24^hi/int/low^ subsets of CD25^+^Foxp3^hCD2+^Rag^GFP+^CD4SP thymocytes (bars indicate mean ± SD of all CpG motifs). The significance was calculated using Kruskal-Wallis with Dunn's test (^*^p < 0.05; ^**^p < 0.01; ^***^p < 0.001).

We had previously demonstrated that tTreg cell maturation, which manifests as a progressive demethylation of the TSDR, is a continuous process that progresses after up-regulation of Foxp3 expression (12). In order to study the dynamics of this maturation process in more detail and in a more precise system, we here made use of transgenic Foxp3^hCD2^xRag1^GFP^ reporter mice, which express green fluorescent protein (GFP) under control of the recombination-activating gene 1 (*Rag1*) promoter (25). In these mice, GFP expression identifies newly generated thymocytes and discriminates them from older and/or re-circulating T cells (28, 29). This is of specific importance as Treg cells were recently shown to re-enter the thymus from the periphery (30), thereby blurring the analysis of tTreg cell development. Here, we first confirmed by flow cytometric analysis that a decrease in CD24 expression, which is associated with thymocyte maturation (12, 31), correlates with a decrease in GFP intensity within the newly generated (Rag^GFP+^) CD4SP thymocyte compartment (Figure S1). Futhermore, flow cytometric analysis of CD24^hi^, CD24^int^ and CD24^low^ subsets among newly generated (Rag^GFP+^) CD25^+^Foxp3^+^ Treg cells (Figure 1A) revealed a significant increase in CD25 expression levels as well as a trend-wise increase in Foxp3^hCD2^ expression from the CD24^hi^ to the CD24^int^ Treg cell subset (Figures 1B,C). In contrast, no difference was observed in expression levels of CD25 and Foxp3^hCD2^ between CD24^int^ and CD24^low^ Treg cell subsets.

The demethylation of Treg cell-specific epigenetic signature genes is a prerequisite for the stable and long-term expression of Foxp3 in Treg cells and their stable suppressive phenotype. This process was reported to be induced already at early stages of Treg cell development within the thymus (11). Additionally, progressive demethylation of the TSDR occurs along maturation of bulk Foxp3^+^ CD4SP thymocytes (12). To gain a more precise insight into the dynamic imprinting of the Treg cell-specific hypomethylation pattern during thymic Treg cell maturation, we FACS-sorted CD24^hi^, CD24^int^, and CD24^low^ subsets of newly generated (Rag^GFP+^) CD25^+^Foxp3^hCD2+^ Treg cells. Subsequently, genomic DNA from these subsets was bisulfite treated and analyzed by pyrosequencing. Interestingly, we observed a successive demethylation of the TSDR, *Eos, Ctla-4*, and *Gitr* correlating with CD24 down-regulation (Figures 1D,E). Intriguingly, *Helios* was already partially demethylated in the CD24^hi^ subset, and no further decrease in its methylation status during Treg cell maturation was observed (Figures 1D,E). Thus, these data suggest that newly generated (Rag^GFP+^) CD25^+^Foxp3^hCD2+^ Treg cells continuously mature within the thymus, observed as increase in CD25 and Foxp3^hCD2^ expression and progressive demethylation of distinct Treg cell-specific epigenetic signature genes.

### Foxp3^+^ TregP Arise at a More Mature Developmental Stage Than CD25^+^ TregP and Display a Partially Demethylated Treg Cell-Specific Epigenetic Signature

**Figure 2:**
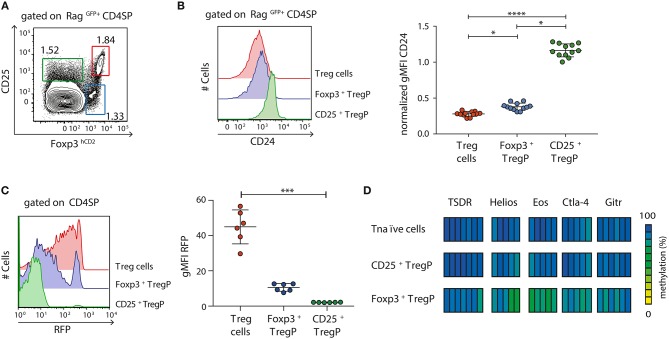
Foxp3^+^ TregP already display a partially demethylated Treg cell-specific epigenetic signature. **(A,B)** Thymocytes were isolated from Foxp3^hCD2^xRag1^GFP^ reporter mice and analyzed by flow cytometry. **(A)** Representative dot plot shows the gating of CD25^+^Foxp3^hCD2−^ (CD25^+^ TregP), CD25^−^Foxp3^hCD2+^ (Foxp3^+^ TregP), and CD25^+^Foxp3^hCD2+^ (Treg cells) cells among Rag^GFP+^CD4SP thymocytes. Numbers specify frequencies of cells in indicated gates. **(B)** (Left) Representative histograms depict CD24 expression among CD25^+^ TregP, Foxp3^+^ TregP and Treg cells. (Right) Scatter plot summarizes the data from three independent experiments and bars indicate mean ± SD. Each symbol represents an individual mouse. The significance was calculated using Kruskal-Wallis with Dunn's test (^*^p < 0.05; ^****^p < 0.0001). Data are given relative to the maximal span (x_max_-x_min_) per experiment. **(C)** Thymocytes were isolated from Foxp3^GFPCre^xROSA26^RFP^ fate-mapping mice and analyzed by flow cytometry. (Left) Representative histograms depict RFP expression among CD25^+^ TregP, Foxp3^+^ TregP and Treg cells. (Right) Scatter plot summarizes the data from three independent experiments and bars indicate mean ± SD. Each symbol represents an individual mouse. The significance was calculated using Kruskal-Wallis with Dunn's test (^***^p < 0.001). **(D)** Thymocytes were isolated from Foxp3^hCD2^ reporter mice, and indicated populations pre-gated on newly generated CD73^low^CD4SP thymocytes were sorted. Genomic DNA was isolated from sorted cells for analysis of the methylation status of TSDR, *Helios, Eos, Ctla-4*, and *Gitr*. Cells were pooled from two independent sorts. Each bar represents an individual CpG motif. Percentage of methylation is color-coded according to the scale.

After having shown that newly generated immature CD24^hi^CD25^+^Foxp3^hCD2+^ Treg cells are already partially demethylated at several Treg cell-specific epigenetic signature genes, we next aimed to assess the methylation status of these signature genes at earlier developmental stages of Treg cells. It was previously reported that tTreg cells arise from either CD25^+^Foxp3^−^ (CD25^+^ TregP) or CD25^−^Foxp3^+^ (Foxp3^+^ TregP) Treg cell precursors (15, 18, 23). Here, we first investigated the maturity of these two different Treg cell precursors and to this end analyzed the expression of CD24 among CD25^+^ TregP and Foxp3^+^ TregP in comparison to newly generated (Rag^GFP+^) CD25^+^Foxp3^+^ Treg cells by flow cytometry (Figures 2A,B). While CD25^+^ TregP displayed a rather immature phenotype with very high CD24 expression levels, Foxp3^+^ TregP showed a significantly decreased CD24 expression almost reaching the levels of CD25^+^Foxp3^+^ Treg cells (Figure 2B). Accordingly, the majority of CD25^+^ TregP was found among CD24^hi^Rag^GFP+^ CD4SP thymocytes, while Foxp3^+^ TregP were mainly enriched in the corresponding CD24^low^ subset (Figure S2). We also analyzed the maturity of Foxp3^+^ TregP in Foxp3^GFPCre^xROSA26^RFP^ mice. In these fate-mapping mice, RFP expression labels Foxp3^+^ cells and their progeny, independently of continuous Foxp3 expression (26). We here found that Foxp3^+^ TregP are mainly constituted of RFP^−/low^ cells when compared to CD25^+^Foxp3^+^ Treg cells, whose majority has accumulated high levels of RFP (Figure 2C). Yet, also a small fraction of Foxp3^+^ TregP expressed high levels of RFP. These cells likely represent the previously described CD25^−^Foxp3^+^ Treg cells re-entering the thymus from the periphery (28). Thus, Foxp3^+^ TregP newly developing in the thymus display an only transient developmental stage precluding accumulation of RFP.

Next, we assessed the methylation pattern of the Treg cell-specific epigenetic signature genes within the two different Treg cell precursors. Pyrosequencing was performed on bisulfite-treated genomic DNA from CD25^+^ TregP and Foxp3^+^ TregP as well as CD25^−^Foxp3^hCD2−^ CD4SP thymocytes (Tnaïve cells) taken as control. Again, only newly generated CD4SP thymocytes, here identified as CD73^low^ cells as described before (23), were included into the analysis (Figure S3). While CD25^+^ TregP displayed a largely methylated Treg cell-specific epigenetic signature comparable to Tnaïve cells, Foxp3^+^ TregP already showed first signs of demethylation particularly in *Eos* and *Helios* (Figure 2D).

Together, these data confirm that Foxp3^+^ TregP arise at a more mature developmental stage when compared to CD25^+^ TregP, which is accompanied by a partial demethylation of the Treg cell-specific epigenetic signature genes. Furthermore, the lack of RFP accumulation in the fate-mapping mice strongly suggests that Foxp3^+^ TregP constitute an only transient developmental stage, in agreement with their precursor nature.

### CD25^+^ TregP and Foxp3^+^ TregP Show Distinct Responsiveness Toward IL-2

**Figure 3:**
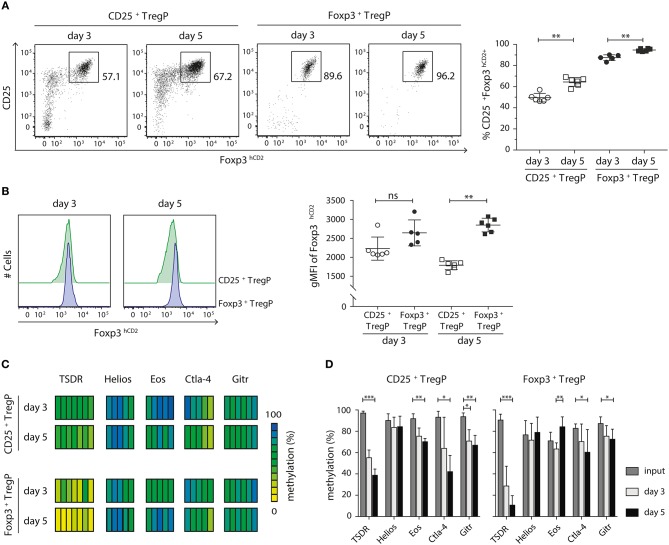
CD25^+^ TregP and Foxp3^+^ TregP show distinct responsiveness toward IL-2. Thymocytes were isolated from Foxp3^hCD2^ reporter mice, CD25^+^ TregP and Foxp3^+^ TregP pre-gated on newly generated CD73^low^CD4SP thymocytes were sorted, and sorted Treg cell precursors were cultured in the presence of rmIL-2. At day 3 or 5 of the culture, CD25^+^Foxp3^hCD2+^ Treg cells were FACS-sorted and analyzed by flow cytometry or prepared for subsequent methylation analysis. **(A)** (Left) Representative dot plots show the gating of CD25^+^Foxp3^hCD2+^ Treg cells from the indicated cultures. Numbers specify frequencies of cells in indicated gates. (Right) Scatter plot summarizes the data from six independent experiments and bars indicate mean ± SD. Each symbol represents an individual experiment. The significance was calculated using Mann-Whitney test (^**^p < 0.01). **(B)** (Left) Representative histograms depict Foxp3^hCD2^ expression among Treg cells sorted from indicated cultures. (Right) Scatter plots summarize the data from six independent experiments and bars indicate mean ± SD. Each symbol represents an individual experiment. The significance was calculated using Mann-Whitney test (ns = not significant; ^**^p < 0.01). **(C,D)** Genomic DNA was isolated from sorted CD25^+^Foxp3^hCD2+^ Treg cells of the indicated cultures for analysis of the methylation status of TSDR, *Helios, Eos, Ctla-4*, and *Gitr*. Cells were pooled from six independent experiments. **(C)** Each bar represents an individual CpG motif. Percentage of methylation is color-coded according to the scale. **(D)** Bar graph summarizes changes in the CpG methylation ratio of the Treg cell-specific epigenetic signature genes in sorted Treg cell precursors (input) and sorted CD25^+^Foxp3^hCD2+^ Treg cells from the indicated cultures (bars indicate mean ± SD of all CpG motifs). The significance was calculated using Kruskal-Wallis with Dunn's test (^*^p < 0.05; ^**^p < 0.01; ^***^p < 0.001).

The two-step model of tTreg cell development proposes that after a first TCR-instructive phase CD25^+^ TregP and Foxp3^+^ TregP are subsequently exclusively dependent on signals derived from cytokines, especially IL-2 (14, 15, 18). As we have shown that CD25^+^ TregP and Foxp3^+^ TregP arise at distinct maturation stages, we next wanted to investigate whether the two Treg cell precursor subsets would also show distinct responsiveness toward IL-2. In order to answer this question, we isolated CD25^+^ TregP and Foxp3^+^ TregP as newly generated CD73^low^ CD4SP thymocytes from Foxp3^hCD2^ reporter mice and cultured them in a minimalistic *in vitro* system for 3 or 5 days in presence of IL-2. Based on previously published findings (15) as well as on preliminary own experiments (Figure S4), a rather high concentration of IL-2 (50 ng/ml) was chosen, which achieved maximal frequencies of CD25^+^Foxp3^+^ Treg cells upon culture of the Treg cell precursors. It is important to note that culture of CD25^+^ TregP and Foxp3^+^ TregP in absence of IL-2 does not lead to any induction of Foxp3 and CD25 expression, respectively (15, 18, 23). At the end of the cultures, developing CD25^+^Foxp3^hCD2+^ Treg cells were FACS-sorted, and further flow cytometric analysis as well as pyrosequencing of bisulfite-treated genomic DNA was performed. In line with previously published observations (18, 23), only a fraction (~50–60%) of CD25^+^ TregP matured into CD25^+^Foxp3^hCD2+^ Treg cells upon stimulation with IL-2 (Figure 3A). In contrast, the majority (85–95%) of Foxp3^+^ TregP up-regulated CD25 expression upon stimulation with IL-2 resulting in CD25^+^Foxp3^hCD2+^ Treg cells (Figure 3A), demonstrating a high responsiveness of Foxp3^+^ TregP to IL-2 signals although they lack expression of the high affinity IL-2 receptor. For both Treg cell precursors, the frequencies of CD25^+^Foxp3^hCD2+^ Treg cells further increased from day 3 to 5 of culture (Figure 3A). Additionally, the expression levels of Foxp3^hCD2^ were elevated in Treg cells arising from Foxp3^+^ TregP when compared to CD25^+^ TregP, particularly at day 5 of culture (Figure 3B). These data indicate a more pronounced maturation of Foxp3^+^ TregP in response to IL-2 when compared to CD25^+^ TregP, which is in line with the observed more mature phenotype of Foxp3^+^ TregP.

Finally, we assessed the methylation status of the Treg cell-specific epigenetic signature genes in CD25^+^Foxp3^hCD2+^ Treg cells generated *in vitro* from the two different Treg cell precursors upon stimulation with IL-2. In any culture, the most pronounced IL-2-induced demethylation was observed at the TSDR (Figures 3C,D). Yet, *Ctla-4*, and *Gitr* were also significantly demethylated in both cultured Treg cell precursors. Interestingly, while cultured CD25^+^ TregP showed a significant demethylation at *Eos*, this epigenetic signature gene was remethylated in Foxp3^+^ TregP cultured for 5 days (Figure 3D). Importanly, the TSDR demethylation was overall more pronounced in cultured Foxp3^+^ TregP when compared to cultured CD25^+^ TregP, reaching almost complete demethylation at day 5 (Figures 3C,D). Together, these results suggest that IL-2 is mainly acting on the demethylation of the TSDR to ensure stable Foxp3 expression.

## Discussion

The majority of CD25^+^Foxp3^+^ Treg cells are known to develop within the thymus, and several recent studies provided insights into the generation and maturation of tTreg cells (13). The two-step model of tTreg cell development proposes that tTreg cells develop from CD25^−^Foxp3^−^ naïve CD4SP thymocytes into CD25^+^Foxp3^+^ Treg cells via a CD25^+^Foxp3^−^ (CD25^+^ TregP) or a CD25^−^Foxp3^+^ (Foxp3^+^ TregP) precursor stage, and these two different Treg cell precursors were recently reported to contribute almost equally to the tTreg cell population (23). In this model, the first developmental step is instructed by TCR signaling and co-stimulation, whereas the second step was shown to depend on signals derived from common γ-chain cytokines, particularly IL-2 (14, 15, 18). Importantly, before their egress from the thymus, CD25^+^Foxp3^+^ Treg cells further mature, shown as progressive demethylation of the TSDR along different maturity stages of Treg cells (12). The establishment of a Treg cell-specific hypomethylation pattern, including the TSDR and additional genes such as *Gitr, Helios, Eos*, and *Ctla-4*, is a prerequisite for the stable suppressive phenotype of Treg cells (9–12). However, specific dynamics of the imprinting of the Treg cell-specific epigenetic signature during tTreg cell development and maturation, and the exact role of IL-2 in directing these processes still remain elusive.

In the present study, we, therefore, performed flow cytometric and methylation analyses on the two Treg cell precursors as well as on Treg cells of different maturation stages. We could demonstrate that CD25^+^Foxp3^+^ Treg cells already display a partially demethylated Treg cell-specific epigenetic signature at the most immature CD24^hi^ stage. These immature Treg cells continuously matured by increasing expression of CD25 and Foxp3 as well as the further progressive establishment of the Treg cell-specific hypomethylation pattern. Importantly, in order to get a precise impression of the events taking place solely within the thymus, we excluded mature Treg cells from our analysis that might have re-circulated from the periphery to the thymus. In addition, we limited our study to “true” Treg cells by analyzing CD25^+^Foxp3^+^ cells rather than the bulk Foxp3^+^ population. This refined way of analysis explains the small discrepancies between the overall lower TSDR methylation level of CD24^hi^, CD24^int^, and CD24^low^ subsets of tTreg cells obtained in the present study compared to previously published results from our group (12).

We could also confirm that Foxp3^+^ TregP arise later in ontogeny and are phenotypically at a more mature developmental stage when compared to CD25^+^ TregP (23). In line with a study from Tai et al., proposing that Foxp3 is lethal for developing thymocytes unless counteracted by cytokine signaling (18), we demonstrated that Foxp3^+^ TregP represent a very transient population. Interestingly, we could show that Foxp3^+^ TregP but not CD25^+^ TregP already display a partial demethylation of the Treg cell-specific epigenetic signature, particularly in *Eos* and *Helios*. These data strongly suggest that at least a part of the Treg cell-specific epigenetic signature is already engraved into the developing tTreg cells before they have entered the very transient Foxp3^+^ TregP stage, in line with the finding that this unique hypomethylation pattern can be fully established even in the absence of Foxp3 expression (11).

Intriguingly, *in vitro* culture of the two Treg cell precursors in the presence of IL-2 mainly resulted in the progressive demethylation of the TSDR with a more pronounced effect seen for Foxp3^+^ TregP. The demethylation of the TSDR was reported to be induced by an active process involving enzymes of the Ten-Eleven-Translocation (Tet) family, which act by iterative oxidation of 5mC to 5hmC (12, 24, 32). In this respect, IL-2 was shown to be required for the maintenance of Tet2 at high levels during tTreg cell development. Tet2 further protects the CpG motifs of the TSDR from re-methylation, leading to stable Foxp3 expression in Treg cells (32, 33). These findings support our observation that cultures of Treg cell precursors with IL-2 resulted in the progressive demethylation of the TSDR. This effect was more pronounced in Treg cells arising from Foxp3^+^ TregP when compared to CD25^+^ TregP. In line with this, Foxp3^+^ TregP developed into CD25^+^Foxp3^+^ Treg cells at increased frequencies, and Treg cells arising from Foxp3^+^ TregP displayed elevated Foxp3 expression levels when compared to Treg cells arising from CD25^+^ TregP. The superior demethylation observed in Treg cells arising from Foxp3^+^ TregP may directly result in these increased Foxp3 expression levels, as IL-2 signaling was shown to stabilize Foxp3 expression in Treg cells by activation of STAT5, which binds directly to the demethylated, open TSDR and enhances Foxp3 expression (34–36).

In contrast to the TSDR demethylation, to the best of our knowledge a direct link between IL-2 signaling and the establishment of the Treg cell-specific hypomethylation pattern at the other epigenetic signature genes has not been reported so far. Interestingly, we here could demonstrate that also *Ctla-4* and *Gitr* get significantly demethylated in both Treg cell precursors upon culture with IL-2. Yet, opposing effects were observed for *Eos*, and *Helios* did not show any signs of demethylation. Our observation that, with the exception of the TSDR in cultured Foxp3^+^ TregP, none of the Treg cell-specific epigenetic signature genes got completely demethylated upon culture with IL-2 implies the requirement for other factors, lacking in the applied minimalistic *in vitro* culture system, for full acquisition of the Treg cell-specific hypomethylation pattern. In this regard, Owen et al. already has shown a differential need of co-stimulatory signals and other cytokines besides IL-2 for CD25^+^ TregP and Foxp3^+^ TregP, in line with their differential localization within the thymus (23).

In conclusion, the results of the present study show that the developmental maturation of tTreg cells is a continuous process, accompanied by the imprinting of the Treg cell-specific epigenetic signature, which endows these tTreg cells with stable Foxp3 expression and stable suppressive properties. The two known tTreg cell precursors display distinct dynamics during the establishment of the Treg cell-specific hypomethylation pattern and further molecular factors involved in this imprinting process need to be elucidated in the future.

## Data Availability Statement

All datasets generated for this study are included in the manuscript/Supplementary Files.

## Author Contributions

SHe, AT, BP, and TM performed the experiments. YK, NO, SF, SHo, and SS discussed and interpreted the data. SHe, AT, and JH designed the research, interpreted the data, and wrote the manuscript.

### Conflict of Interest

The authors declare that the research was conducted in the absence of any commercial or financial relationships that could be construed as a potential conflict of interest. The reviewer BS and handling editor declared their shared affiliation at the time of review.
