# Supplementary material for: Dynamic Imprinting of the Treg Cell-Specific Epigenetic Signature in Developing Thymic Regulatory T Cells
Source: Front Immunol. 2019 Oct 11;10:2382. doi: 10.3389/fimmu.2019.02382 (PMC6797672; doi:10.3389/fimmu.2019.02382)
Supplement: Supplementary file 1 [file Data_Sheet_1.pdf]

Supplementary Material

Dynamic imprinting of the Treg cell-specific epigenetic signature in developing thymic regulatory T cells

Susanne Herppich, Aras Toker, Beate Pietzsch, Yohko Kitagawa, Naganari Ohkura, Takahisa Miyao, Stefan Floess, Shohei Hori, Shimon Sakaguchi, Jochen Huehn

Supplementary Figures

Figure S1

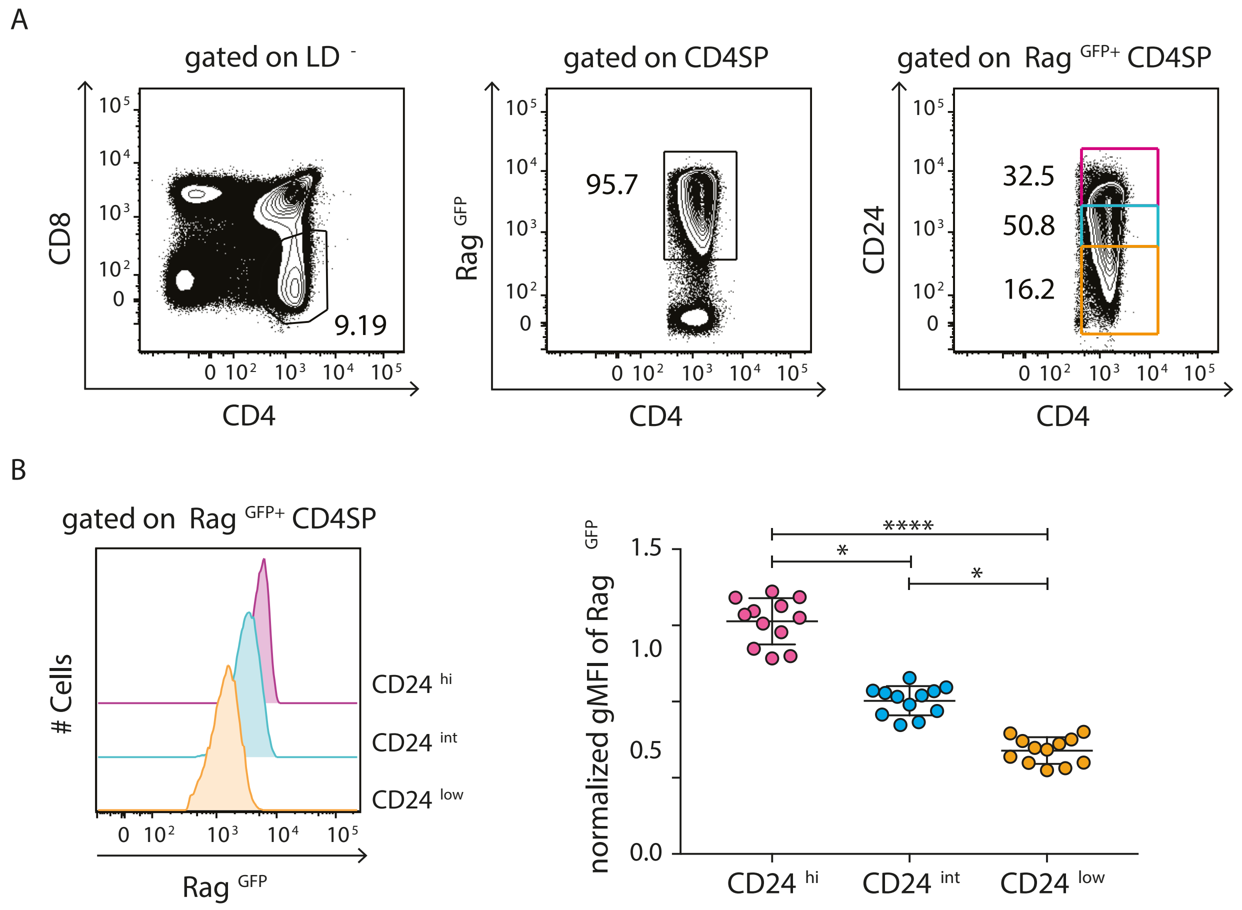

Figure S1. Downregulation of CD24 correlates with downregulation of Rag^{GFP}, indicating maturation of thymocytes. Thymocytes were isolated from Foxp3^{hCD2}xRag1^{GFP} reporter mice and analyzed by flow cytometry. **(A)** Representative dot plots show the gating of CD4SP thymocytes among living cells (LD^{-}, LIVE/DEAD^{-}), Rag^{GFP+} cells among CD4SP thymocytes, and CD24$^{hi/int/low}$ cells among Rag^{GFP+}CD4SP thymocytes. Numbers specify frequencies of cells in indicated gates. **(B)** (Left) Representative histograms depict Rag^{GFP} expression among CD24$^{hi/int/low}$ subsets of Rag^{GFP+}CD4SP thymocytes. (Right) Scatter plot summarizes the data from three independent experiments and bars indicate mean ± SD. Each symbol represents an individual mouse. The significance was calculated using Kruskal-Wallis with Dunn's test (*$p < 0.05$; ****$p < 0.0001$). Data are given relative to the maximal span (x_{max}-x_{min}) per experiment.

Figure S2

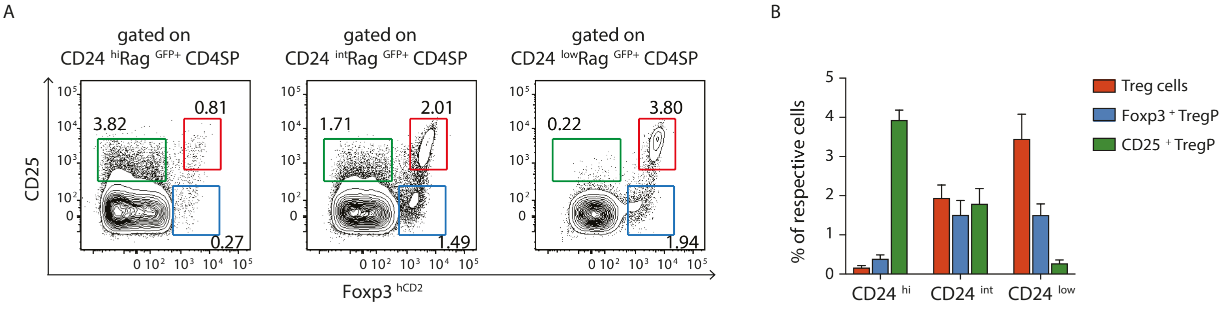

Figure S2. CD25$^+$ TregP, Foxp3$^+$ TregP and Treg cells arise at distinct maturation stages. Thymocytes were isolated from Foxp3^{hCD2}xRag1^{GFP} reporter mice and analyzed by flow cytometry. **(A)** Representative dot plots show the gating of CD25$^+$Foxp3^{hCD2-} (CD25$^+$ TregP, green), CD25$^-$Foxp3^{hCD2+} (Foxp3$^+$ TregP, blue) and CD25$^+$Foxp3^{hCD2+} (Treg cells, red) cells among CD24$^{hi/int/low}$ subsets of Rag^{GFP+}CD4SP thymocytes. Numbers specify frequencies of cells in indicated gates. **(B)** Scatter plot summarizes the data from three independent experiments and bars indicate mean ± SD.

Figure S3

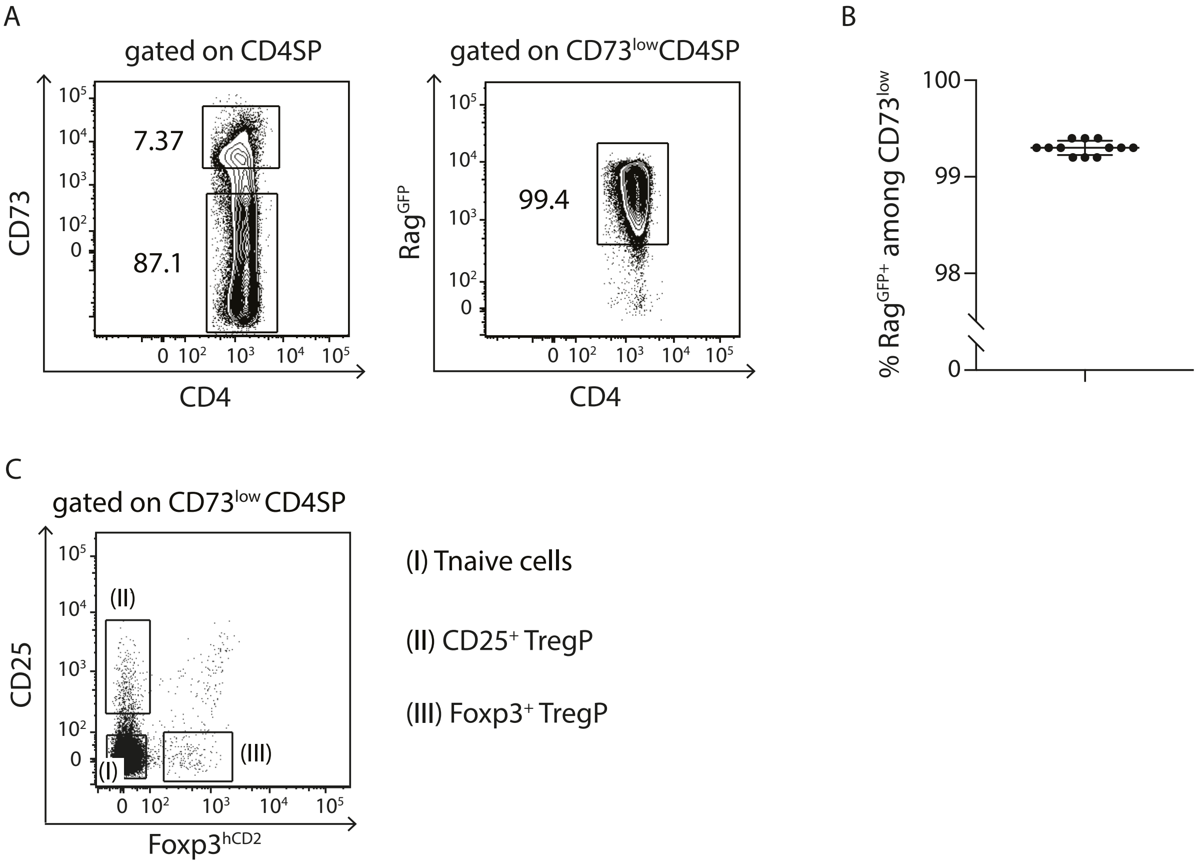

Figure S3. CD73^{low} cells resemble newly generated thymocytes. (A, B) Thymocytes were isolated from Foxp3^{hCD2}xRag1^{GFP} reporter mice and analyzed by flow cytometry. **(A)** Representative dot plots show the gating of CD73^{hi} and CD73^{low} cells among CD4SP thymocytes pre-gated on living cells as well as Rag^{GFP+} cells among CD73^{low}CD4SP thymocytes. Numbers specify frequencies of cells in indicated gates. **(B)** Scatter plot summarizes the data from three independent experiments and bars indicate mean ± SD. Each symbol represents an individual mouse. **(C)** Sorting strategy for indicated populations from Foxp3^{hCD2} reporter mice pre-gated on CD73^{low}CD4SP thymocytes.

Figure S4

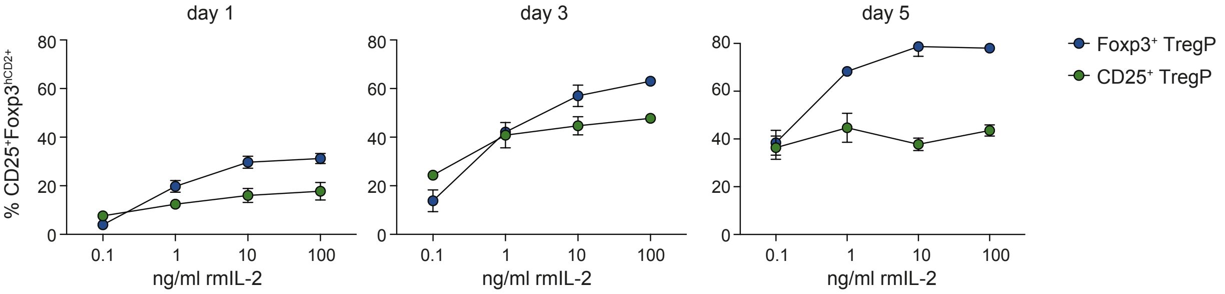

Figure S4. Culture of CD25⁺ TregP and Foxp3⁺ TregP with different concentrations of rmIL-2. Thymocytes were isolated from Foxp3^{hCD2} reporter mice, CD25⁺ TregP and Foxp3⁺ TregP were sorted, and sorted Treg cell precursors were cultured in the presence of indicated doses of rmIL-2. At day 1, 3 or 5 of the culture, frequencies of CD25^{+}Foxp3^{hCD2+} Treg cells were analyzed by flow cytometry. Scatter plot summarizes the data from one experiment and bars indicate mean ± SD of technical replicates.
